# Lower bioelectrical impedance phase angle is associated with COPD and is a marker for increased risks in elderly COPD patients

**DOI:** 10.3389/fmed.2025.1703621

**Published:** 2026-01-13

**Authors:** JinHua Qian, Min Xu, ZhaoXi Zhang, YuJie Wu, GuoQing Wang, XiaoYun Fan

**Affiliations:** 1 Department of Geriatric Respiratory and Critical Care Medicine, The First Affiliated Hospital of Anhui Medical University, Hefei, Anhui, China; 2 Department of Geriatric Medicine, Huangshan city People’s Hospital, Huangshan, China

**Keywords:** PHA, COPD, grip strength, knee extension strength, BIA

## Abstract

**Introduction:**

The phase angle (PhA), derived from bioelectrical impedance analysis (BIA), serves as an indicator of cellular health and body composition. While associated with muscle strength and exercise capacity in various conditions, its clinical relevance in chronic obstructive pulmonary disease (COPD) requires further characterization. This study aimed to evaluate the relationship between PhA, muscle strength, and physical function among individuals with COPD.

**Methods:**

Between June 2024 and August 2025, 112 male patients with COPD and 20 healthy male controls were enrolled in this cross-sectional study. Assessments included pulmonary function, body composition via BIA, handgrip strength, knee extension strength, walking speed, and other clinical indicators. Relationships were analyzed using multivariable linear and least absolute shrinkage and selection operator (LASSO) regression models.

**Results:**

PhA values were significantly lower in COPD patients than in healthy controls. Stratification of COPD patients by PhA revealed that a lower PhA was associated with progressively worse muscle strength, exercise capacity, and other clinical markers. Multivariable linear regression analyses demonstrated that a lower PhA was independently associated with slower walking speed (*β* = 0.061, *p* < 0.001) and reduced knee extension strength (*β* = 1.15, *p* = 0.002). Furthermore, PhA was selected as a key predictor in a prognostic model for severe physical impairment derived from the LASSO regression analysis.

**Conclusion:**

In this cross-sectional study, a lower PhA is independently associated with muscle weakness and impaired physical performance in men with COPD. These findings suggest that PhA may serve as a useful biomarker for assessing nutritional and functional status in this population. However, the cross-sectional design precludes causal inference, and the diagnostic utility of PhA for COPD itself is not established.

## Introduction

Chronic obstructive pulmonary disease (COPD) is a prevalent respiratory condition primarily caused by long-term tobacco use, environmental pollutants, and recurrent respiratory infections (1, 2). In China, over 27% of elderly individuals are affected by COPD (3), which ranks as the fourth leading cause of death globally (4). Beyond impaired lung function, patients frequently experience systemic manifestations, including anemia, depression, sarcopenia, malnutrition, and cardiovascular comorbidities (5, 6). Malnutrition further impairs respiratory muscle function (7, 8), potentially leading to respiratory failure and increased mortality (9, 10).

Bioelectrical impedance analysis (BIA) is a recognized technique for assessing body composition and diagnosing sarcopenia (7, 8). The phase angle (PhA), calculated directly from resistance (R) and reactance (Xc) without estimation equations (11), serves as a marker of cellular membrane integrity and viability (12). Higher PhA values suggest better membrane integrity and functional capacity (13). PhA levels are typically lower in women than in men, and they decrease as they age (14, 15). PhA also reflects muscle mass, strength, fat-free mass (FFM), and hydration status, underscoring its utility in sarcopenia diagnosis (16). Clinically, PhA holds prognostic value for older adults and patients with COPD, cancer, or those undergoing hemodialysis. In COPD populations, PhA inversely correlates with poor prognostic markers but shows a positive association between fat-free mass and physical performance (17). Notably, PhA proves to be a more robust metric than fat-free mass alone, as it correlates with exercise tolerance and the *body mass index, airflow obstruction, dyspnea, and exercise capacity index* (BODE) index independent of fat-free mass (18). Thus, PhA is a valuable BIA-derived indicator of nutritional status and cellular health (19). Despite existing studies linking BIA-derived PhA to nutritional status, data specifically focusing on elderly COPD patients remain limited (20).

Given the established relevance of PhA in various diseases, including cancer, hepatic fibrosis, heart failure, and renal failure (11, 13), this study aimed to investigate whether PhA can serve as an indicator of systemic involvement in COPD. Demonstrating such an association would extend the clinical significance of PhA beyond its role in skeletal muscle assessment, positioning it as a comprehensive marker of systemic impairment in COPD.

## Materials and methods

### Participants

This single-center, observational, cross-sectional study was conducted at our hospital and was approved by the institutional review board and ethics committee (Approval number: 2024-A-009, approval date: June 10, 2024). All participants provided written informed consent, and the study was conducted in accordance with the Declaration of Helsinki.

From June 2024 and August 2025, we recruited 112 male patients with clinically stable COPD undergoing routine follow-up, along with 20 healthy male volunteers. The exclusion criteria were as follows: (1) COPD exacerbation within the past month, (2) contraindications to BIA such as cardiac pacemaker implantation, (3) incomplete data, or (4) unwillingness to participate. Participants were informed of their right to withdraw at any time, and only routine clinical data were collected without additional invasive procedures.

### Sample size consideration

The sample size of 112 COPD patients and 20 controls was determined by the available cohort during the study period. A post-hoc precision analysis was performed for the primary correlation between phase angle (PhA) and the 6-min walk distance (6MWD), which yielded a correlation coefficient of *r* = 0.69. For this effect size, with an alpha error of 0.05 and a power of 90%, the required sample size would be approximately 25 participants. Our sample of 112 patients, therefore, provides ample statistical power to detect the observed association and allows for precise estimation, as evidenced by the narrow confidence intervals around our correlation and regression coefficients.

### Blood test

The following parameters were retrieved from electronic medical records: serum albumin, total lymphocyte count, total cholesterol, hemoglobin, and C-reactive protein.

### Body composition

Body composition was assessed via whole-body bioelectrical impedance analysis (BIA) using a Bodystat Quadscan 4000 analyzer (Bodystat Ltd., Isle of Man, United Kingdom) (21). The measured parameters included fat-free mass (FFM), from which the fat-free mass index (FFMI, FFM/height^2^ in kg/m^2^) and skeletal muscle mass index (SMI) were derived. PhA and the extracellular water-to-total body water ratio (ECW/TBW) were also obtained (22).

### Bioelectrical impedance

Prior to measurement, participants fasted for at least 1.5 h, emptied their bladders, and refrained from moderate-to-vigorous physical activity for 12 h. Following a 15-min rest period in a supine position on a non-conductive surface, the BIA was performed. The measurement used a tetrapolar electrode configuration on the dominant side of the body. An alternating current of 800 μA at a frequency of 50 kHz was applied. Surface electrodes (Bodystat Ltd., Isle of Man, United Kingdom) were placed at standard anatomical sites: on the dorsum of the hand proximal to the metacarpal-phalangeal joint, on the wrist at the midline between the distal prominences of the radius and ulna, on the foot between the medial and lateral malleoli at the ankle, and on the dorsum of the foot proximal to the metatarsal-phalangeal joint.

Resistance (R) and reactance (Xc) were measured directly by the device. The intraday measurement variability was below 2% for R and 3.5% for Xc. The PhA was computed at 50 kHz using the following formula: PhA (°) = arctangent (Xc/R) × (180/*π*) with dedicated software (Bodystat Ltd., Isle of Man, United Kingdom). PhA values were classified as low or normal based on age-, sex-, and body mass index (BMI)-stratified reference percentiles from a large healthy cohort (*n* = 214,732) (23). The standardized phase angle was calculated as follows: (observed PhA − mean reference PhA)/SD of reference PhA. The PhA was analyzed both as a raw variable (units: degrees) and as a *z*-standardized score. The *z*-standardization was performed to facilitate interpretation relative to a healthy reference population and was used in the studies. We applied the reference values from Barbosa-Silva et al.’s study, which provide mean and standard deviation values for PhA stratified by sex and age groups in a large healthy sample (*n* = 1,967). For each participant, the *z*-score was calculated as follows: (observed PhA − mean reference PhA for their sex and age group)/standard deviation of the reference PhA for their sex and age group.

Fat-free mass (FFM) was estimated using sex-specific regression equations (24, 25): For women, FFM was calculated as follows: FFM = 7.610 + 0.474 × (height^2^/R) + 0.184 × weight, and for men, FFM was calculated as follows: FFM = 0.383 + 0.465 × (height^2^/R) + 0.213 × weight.

### Patient background

Data collected from medical records included: age, chronic obstructive lung disease (GOLD) stage (I: mild, forced expiratory volume in 1 second (FEV₁) ≥ 80% predicted; II: moderate, 50% ≤ FEV₁ < 80%; III: severe, 30% ≤ FEV₁ < 50%; and IV: very severe, FEV₁ < 30% predicted), modified Medical Research Council (mMRC) dyspnea score, exacerbation history in the previous year, use of home oxygen therapy, comorbidities, and medications (26).

### Pulmonary function

Pulmonary function was assessed using the CHESTAC-8800 system (Chest Co., Ltd., Tokyo, Japan) following standard guidelines (27). Forced vital capacity (FVC), FEV₁, and FEV₁/FVC ratio were measured via forced spirometry (28). Inspiratory capacity (IC) was determined using the slow vital capacity technique. Diffusing capacity for carbon monoxide (DLCO) was measured using the single-breath method (29).

Predicted values for FVC and FEV₁ were derived from the 1988 Chinese national spirometric reference equations, which were established from a large sample (*n* = 7,115) across six major administrative regions in China and were recommended by the Chinese Expert Consensus on Adult Pulmonary Function Diagnosis (2022) for their applicability to the Chinese population (27, 30, 31). For DLCO, predicted values were calculated using the 2011 revised reference equations specifically derived for Chinese adults (28, 32–34). The use of these population-specific equations minimizes potential bias in the derived percentage of predicted values.

### Physical function assessment

The 6-min walk test (6MWT) was administered according to the American Thoracic Society guidelines (35), and the 6-min walk distance (6MWD) was recorded. Usual walking speed was assessed as a separate, direct measure of gait speed. Participants were instructed to walk at their usual, comfortable pace over a straight, flat distance of 4 m. The time taken to cover the central 3 m (to exclude acceleration and deceleration phases) was measured using a stopwatch. Walking speed was then calculated in meters per second (m/s). This short walk test is a widely used and reliable measure of usual gait speed in older and clinical populations. Walking speed was calculated based on the 6MWD. Handgrip strength was measured using a Smedley-type dynamometer (Grip-D; Takei Scientific Instruments Co., Ltd., Niigata, Japan), with the highest reading used (36). Knee extension strength was assessed using a μTas F-1 system (Anima Co., Ltd., Tokyo, Japan), and the maximum value was recorded.

### Prognostic nomogram analysis

Least absolute shrinkage and selection operator (LASSO) regression analysis was used for variable selection and dimensionality reduction. Non-zero coefficients from the LASSO model were included in a multivariable logistic regression analysis to develop a predictive nomogram. Model calibration was evaluated using calibration curves, and discriminative ability was assessed using Harrell’s C-index. A decision curve analysis was used to evaluate clinical utility, and diagnostic performance was determined using the receiver operating characteristic curve analysis (AUC) in the training and validation cohorts (37–39).

### Statistical analysis

Non-normally distributed data are presented as median [interquartile range (IQR)] or proportions with 95% confidence intervals (CIs). Spearman’s correlation coefficient was used to evaluate associations between PhA, FFM, FFMI, and other variables. Group comparisons (low vs. normal PhA or FFMI) were performed using the Mann–Whitney *U*-test. Multivariable regression analyses were conducted to identify determinants of square-root-transformed indicators, including incremental shuttle walk (ISW) distance, 4-m gait speed (4MGS), 5-repetition sit-to-stand test (5STS), and Age, Dyspnea, and Airflow Obstruction Index Score (ADO) index scores. Independent variables included PhA, FFMI, age, sex, BMI, percent predicted FEV₁, mMRC dyspnea score, quadriceps maximal voluntary contraction (QMVC), and the Charlson comorbidity index. For the ADO model, age, percent predicted FEV₁, and mMRC score were excluded, as they are components of this composite index. The variables were checked for collinearity (*r* < 0.5) and included or excluded through a stepwise selection process (entry *p* < 0.05, removal *p* ≥ 0.10).

## Results

A total of 112 male patients diagnosed with COPD and 20 healthy male volunteers were enrolled in this study. The demographic and clinical characteristics of the participants are summarized in Table 1. The mean age of the COPD group was 75.12 ± 1.27 years, which was comparable to that of the control group (74.23 ± 3.99 years). The average body mass index (BMI) was also similar between the two groups (COPD: 23.11 ± 2.61 vs. control: 23.58 ± 3.26). However, significant differences were observed in body composition and functional parameters. The fat-free mass index (FFMI) was notably lower in the COPD group (16.24 ± 2.64) than in the control group (18.25 ± 3.25). Furthermore, the COPD group exhibited significant reductions in the skeletal muscle mass index (SMI), PhA, and walking speed. The prevalence of sarcopenia and Global Leadership Initiative on Malnutrition (GLIM)-defined malnutrition was also substantially higher among COPD patients.

**Table 1:** Characteristics of patients.

Characteristers	Control (*N* = 20)	COPD (*N* = 112)	*p*-value
Age (years)	74.23 ± 3.99	75.12 ± 1.27	0.321
GOLD (I/II/III/IV), *n* (%)	&	19/62/17/14	&
mMRC (0/1/2/3/4), *n* (%)	&	12/45/36/19	&
Exacerbation frequency (0/1/2+), *n* (%)	&	74/14/24	&
Use of home oxygen therapy, *n* (%)	&	21	&
Comorbidities (0/1/2/3+), *n* (%)	&	5/38/44/25	&
Medication, *n* (%)			&
Steroid ICS/prednisone	&	102/10	&
Dyslipidemic drug	&	52	&
SGLT2 inhibitors	&	9	&
BMI (kg/m^2^)	23.58 ± 3.26	23.11 ± 2.61	0.065
FFMI (kg/m^2^)	18.25 ± 3.25	16.24 ± 2.64	0.015
SMI (kg/m^2^)	8.31 ± 0.89	6.02 ± 0.77	0.022
PhA (°)	5.35 ± 1.22	4.08 ± 0.48	0.033
ECW/TBW	0.39 ± 0.02	0.38 ± 0.02	0.614
Sarcopenia, *n* (%)	2	42	0.021
GLIM malnutrition, *n* (%)	0	52	0.042
MUST (0/1/2+), *n* (%)	20/0/0	85/20/7	0.215
FEV1 (% predicted)	&	58.15 ± 15.84	&
FVC (% predicted)	&	87.48 ± 11.25	&
FEV1/FVC (%)	&	50.26 ± 9.58	&
DLCO (% predicted)	&	38.22 ± 6.68	&
IC (L)	&	2.26 ± 0.45	&
Grip strength (kg)	35.18 ± 7.26	33.28 ± 7.05	0.66
Walking speed (m/s)	1.58 ± 0.21	1.00 ± 0.21	0.002
6MWD (m)	&	388.62 ± 120.28	&
Knee extension strength (kgf/kg)	&	0.55 ± 0.08	&
Alb (g/dL)	&	4.19 ± 0.52	&
TLC (/μL)	&	1542.69 ± 152.62	&
T-Cho (mg/dL)	&	181.64 ± 25.88	&
Hb (g/dL)	&	14.28 ± 2.67	&
CRP (mg/dL)	&	0.18 ± 0.02	&

BMI, body mass index; FFMI, fat-free mass index; SMI, skeletal muscle mass index; PhA, phase angle; ECW/TBW, extracellular water-to-total body water ratio; GOLD, Global Initiative for Chronic Obstructive Lung Disease; mMRC, modified Medical Research Council; ICS, inhaled corticosteroid; SGLT2, sodium–glucose cotransporter-2; GLIM, Global Leadership Initiative on Malnutrition; MUST, Malnutrition Universal Screening Tool; FEV₁, forced expiratory volume in 1 second; FVC, forced vital capacity; DLCO, diffusing capacity for carbon monoxide; IC, inspiratory capacity; 6MWD, 6-min walk distance; Alb, albumin; TLC, total lymphocyte count; T-Cho, total cholesterol; Hb, hemoglobin; CRP, C-reactive protein.

When patients were stratified based on PhA values (Table 2), the subgroup with lower PhA demonstrated a significantly higher extracellular water-to-total body water ratio (ECW/TBW) than the higher PhA subgroup. This lower PhA subgroup also exhibited markedly reduced values for multiple parameters, including BMI, FFMI, SMI, lung function parameters (FEV₁, FEV₁/FVC, DLCO, and IC), muscle strength (handgrip strength and knee extension strength), functional capacity (walking speed and 6MWD), and biochemical markers (total cholesterol and hemoglobin).

**Table 2:** Patient data comparison between the low- and high-PhA groups.

Characteristers	Low group (*n* = 54)	High group (*n* = 58)	*p*-value
Age (years)	78.08 ± 2.22	72.18 ± 2.36	0.001
GOLD (I/II/III/IV), *n* (%)	8/24/11/11	11/38/6/3	0.524
mMRC (0/1/2/3/4), *n* (%)	5/20/21/8	7/25/15/11	0.245
Exacerbation frequency (0/1/2+), *n* (%)	0034/8/12	0030/6/12	0.011
Use of home oxygen therapy, *n* (%)	11	10	0.066
Comorbidities (0/1/2/3+), *n* (%)	2/20/18/14	3/18/26/11	0.157
Medication, *n* (%)			
Steroid ICS/prednisone	0051/3	0051/7	0.264
Dyslipidemic drug	32	20	0.581
SGLT2 inhibitors	5	4	0.154
BMI (kg/m^2^)	22.61 ± 3.69	23.95 ± 2.21	0.002
FFMI (kg/m^2^)	14.89 ± 2.08	17.25 ± 1.77	0.012
SMI (kg/m^2^)	6.02 ± 0.45	7.15 ± 0.85	0.032
PhA (°)	4.11 ± 0.24	5.65 ± 0.24	0.001
ECW/TBW	0.38 ± 0.08	0.30 ± 0.02	0.015
Sarcopenia, *n* (%)	24	21	0.325
GLIM malnutrition, *n* (%)	30	22	0.331
MUST (0/1/2+), *n* (%)	0049/3/2	0036/17/5	0.251
FEV1 (% predicted)	52.61 ± 15.64	66.25 ± 20.15	0.002
FVC (% predicted)	82.67 ± 6.85	98.52 ± 18.55	0.058
FEV1/FVC (%)	47.58 ± 12.22	55.62 ± 9.27	0.023
DLCO (% predicted)	32.02 ± 5.88	39.95 ± 7.48	0.02
IC (L)	1.98 ± 0.52	2.35 ± 0.48	0.032
Grip strength (kg)	24.28 ± 6.64	35.94 ± 9.56	0.001
Walking speed (m/s)	0.95 ± 0.05	1.15 ± 0.04	0.032
6MWD (m)	352.61 ± 110.58	469.84 ± 102.48	0.011
Knee extension strength (kgf/kg)	0.40 ± 0.05	0.59 ± 0.08	0.022
Alb (g/dL)	4.15 ± 0.65	4.20 ± 0.48	0.185
TLC (/μL)	1562.35 ± 142.61	1,566 ± 156.35	0.299
T-Cho (mg/dL)	156.28 ± 15.94	199.34 ± 19.65	0.026
Hb (g/dL)	12.65 ± 1.98	14.89 ± 2.25	0.011
CRP (mg/dL)	0.17 ± 0.06	0.18 ± 0.09	0.095

BMI, body mass index; FFMI, fat-free mass index; SMI, skeletal muscle mass index; PhA, phase angle; ECW/TBW, extracellular water-to-total body water ratio; GOLD, Global Initiative for Chronic Obstructive Lung Disease; mMRC, modified Medical Research Council; ICS, inhaled corticosteroid; SGLT2, sodium–glucose cotransporter-2; GLIM, Global Leadership Initiative on Malnutrition; MUST, Malnutrition Universal Screening Tool; FEV₁, forced expiratory volume in 1 second; FVC, forced vital capacity; DLCO, diffusing capacity for carbon monoxide; IC, inspiratory capacity; 6MWD, 6-min walk distance; Alb, albumin; TLC, total lymphocyte count; T-Cho, total cholesterol; Hb, hemoglobin; CRP, C-reactive protein.

Correlation analyses confirmed these associations (Table 3). The PhA showed statistically significant positive correlations with FFMI (*r* = 0.44, *p* = 0.01), SMI (*r* = 0.36, *p* = 0.02), lung function (FEV₁%: *r* = 0.25, *p* = 0.02; FVC: *r* = 0.34, *p* = 0.01; FEV₁/FVC: *r* = 0.62, *p* = 0.02), and physical function (handgrip strength: *r* = 0.25, *p* = 0.03; walking speed: *r* = 0.22, *p* = 0.02; 6MWD: *r* = 0.69, *p* = 0.03).

**Table 3:** Correlation between PhA and indicators of sarcopenia and malnutrition in patients with COPD.

Characteristers	*r*	*rs*	*p*-value
BMI (kg/m^2^)	0.25	−0.25	0.26
FFMI (kg/m^2^)	0.44	−0.15	**0.01**
SMI (kg/m^2^)	0.36	−0.35	**0.02**
FEV1 (% predicted)	0.25	−0.25	**0.02**
FVC (% predicted)	0.34	−0.44	**0.01**
FEV1/FVC (%)	0.62	−0.25	**0.02**
DLCO (% predicted)	0.18	−0.28	0.32
Grip strength (kg)	0.25	−0.11	0.03
Walking speed (m/s)	0.22	−0.32	0.02
6MWD (m)	0.69	−0.58	0.03
Knee extension strength (kgf/kg)	0.35	−0.48	0.18
Alb (g/dL)	0.34	0.15	0.24
TLC (/μL)	0.28	0.24	0.29

BMI, body mass index; FFMI, fat-free mass index; SMI, skeletal muscle mass index; FEV₁, forced expiratory volume in 1 second; FVC, forced vital capacity; DLCO, diffusing capacity for carbon monoxide; 6MWD, 6-min walk distance; Alb, albumin; TLC, total lymphocyte count.

### Independent association between the PhA and physical function

Independent associations between PhA and key physical function parameters were assessed using multivariable linear regression models, after adjusting for age, sex, BMI, and comorbidities. As shown in Table 4, a one-degree increase in the PhA was significantly associated with a 0.061 m/s increase in walking speed (*β* = 0.061, 95% CI: 0.035–0.085, *p* < 0.001). Similarly, for knee extension strength, a one-degree increase in the PhA was associated with a 1.15 kg increase in strength (*β* = 1.15, 95% CI: 1.05–1.62, *p* = 0.002).

**Table 4:** Multivariate logistic regression analysis with sarcopenia and malnutrition as the dependent variable in patients with COPD.

Multivariate	*β* coefficient	Standard errors	95% CI	*p*-value
Model 1 [model for walking speed (m/s)]				
Phase angle (°)	0.061	0.013	0.11–0.85	0.022
Age (years)	0.022	0.041	0.98–1.65	0.069
Comorbidities (0/1/2/3+), *n* (%)	0.041	0.062	0.98–1.35	0.251
FVC (% predicted)	0.066	0.04	0.44–0.95	0.112
Grip strength (kg)	0.011	0.44	0.32–0.85	0.012
Walking speed (m/s)	&	&	&	&
6MWD (m)	0.018	0.068	0.21–0.69	0.001
Knee extension strength (kgf/kg)	0.032	0.022	1.05–1.62	0.015
Model 2 [model for knee extension strength (kg)]				
Phase angle (1°)	0.028	0.011	0.12–0.85	0.032
Age (years)	0.025	0.051	0.89–1.65	0.482
Comorbidities (0/1/2/3+), *n* (%)	0.015	0.015	0.94–1.22	0.325
FVC (% predicted)	0.0058	0.028	0.41–0.69	0.132
Grip strength (kg)	0.154	0.032	0.48–0.95	0.024
Walking speed (m/s)	0.024	0.021	0.35–0.85	0.015
6MWD (m)	0.017	0.002	0.88–1.25	0.031
Knee extension strength (kgf/kg)	&	&	&	&

6MWD, 6-min walk distance; FVC, forced vital capacity.

### Development of a prognostic model for severe physical impairment

**Figure 1:**
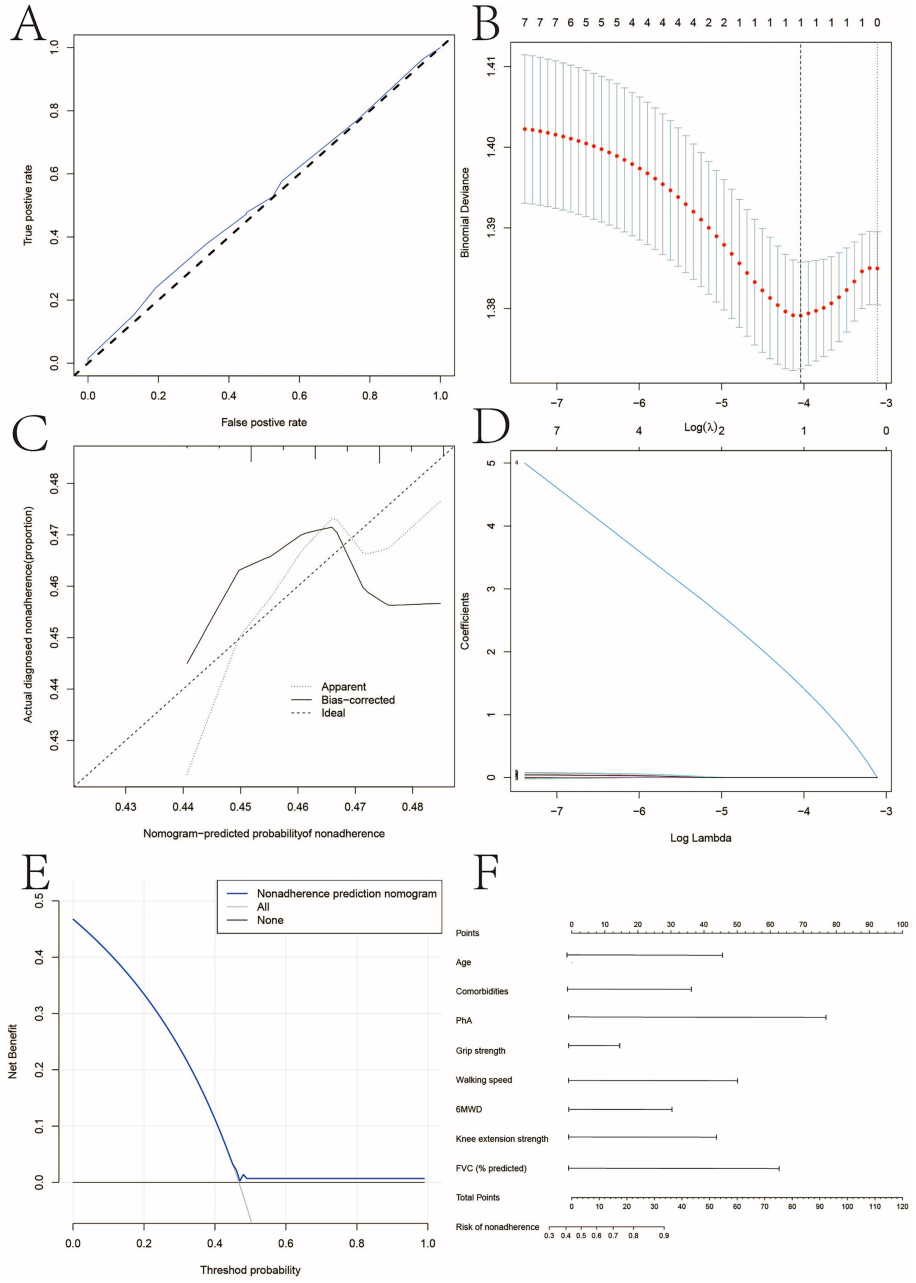
Prognostic nomogram for predicting the probability of severe physical impairment in COPD patients. **(A)** Calibration curves of the non-adherence nomogram prediction in the cohort. **(B)** Optimal parameter (lambda) selection in the LASSO model uses fivefold cross-validation using minimal criteria. **(C)** Decision curve analysis for the non-adherence nomogram. **(D)** LASSO coefficient profiles of the 22 characteristics. **(E)** Decision curve analysis for the non-adherence nomogram. **(F)** A vertical line indicates the optimum lambda value from fivefold cross-validation, yielding five features with non-zero coefficients. BMI, body mass index; FFMI, fat-free mass index; SMI, skeletal muscle mass index; PhA, phase angle; ECW/TBW, extracellular water-to-total body water ratio; FEV₁%, percent predicted forced expiratory volume in 1 second; 6MWD, 6-min walk distance. Data are presented as mean ± standard deviation.

To develop a clinically applicable tool for identifying patients at high risk, we constructed a prognostic model for “severe physical impairment,” defined as the coexistence of low handgrip strength and low 6-min walk distance. A LASSO regression analysis, applied to an initial set of 20 variables, selected five key predictors: age, number of comorbidities, PhA, handgrip strength, and forced vital capacity (FVC). A nomogram was created based on these predictors to estimate individual patient risk (Figure 1).

The model was subjected to rigorous internal validation using the 0.632 + bootstrap method (1,000 resamples) to correct for over-optimism. The validated model demonstrated good discriminative ability, with a bootstrap-corrected C-index of 0.81 (95% CI: 0.75–0.87). The calibration curve showed acceptable agreement between predicted probabilities and observed outcomes (Figure 1A). The decision curve analysis (Figure 1B) confirmed the clinical utility of the nomogram, indicating that its use for risk stratification provides a positive net benefit across a wide range of decision thresholds.

## Discussion

Globally, respiratory diseases often receive comparatively less attention and funding relative to their significant contribution to morbidity and mortality (9). Chronic obstructive pulmonary disease (COPD), in particular, is a major public health issue and a persistent challenge for healthcare systems in the 21st century (40). In our cohort, we observed a significantly lower PhA in COPD patients than in healthy controls. Subsequent stratification of COPD patients based on PhA, followed by multivariate and LASSO regression analyses, established PhA as an independent predictor of handgrip strength and knee extension strength in this population.

The PhA, a raw parameter derived from the bioelectrical impedance analysis (BIA), is increasingly used to assess malnutrition across various diseases, including respiratory conditions such as COPD (41, 42). COPD patients with lower PhA are typically older and more likely to experience hypoxia and hypercapnia. Those with more severe disease exhibit reduced body cell mass, significant skeletal muscle wasting, and impaired gas exchange (8, 43). The relationship between PhA and all-cause mortality in COPD patients has been demonstrated using the Cox regression analysis, the Kaplan–Meier analysis, and log-rank test (44, 45). Furthermore, PhA serves as a marker for the role of malnutrition in idiopathic pulmonary fibrosis (IPF), independent of body weight. It correlates with reduced muscle mass in these patients, which impairs their physical strength and exercise capacity and adversely affects prognosis (46). Our research also confirmed a substantial reduction in PhA among individuals with COPD, reinforcing its value as a crucial biomarker.

Within health assessment tools, walking pace (WP) and hand grip strength (HGS) are fundamental metrics that correlate widely with various health outcomes. Numerous studies demonstrated that poorer muscle function is associated with higher mortality and morbidity (47, 48). For example, a 24-year follow-up study of 1,142,599 adolescent male individuals (aged 16–19 years) found that reduced muscular strength was associated with increased all-cause and cardiovascular mortality, but not with cancer mortality (49). Recent data from the Prospective Urban–Rural Epidemiology (PURE) study, which included 139,691 adults aged 35–70 years with a 4-year follow-up, demonstrated an inverse relationship between grip strength and all-cause, non-cardiovascular, and cardiovascular mortality. However, no significant association was observed with respiratory diseases, including COPD (50). A bidirectional causal relationship has been suggested between usual walking pace and COPD risk, whereas a decrease in right-hand grip strength exhibits a unidirectional relationship with an increased incidence of COPD (51). HGS is also linked to peak inspiratory flow rate (PIFR) in clinically stable COPD patients, particularly in those with significant symptoms but infrequent exacerbations. Threshold values of HGS associated with poor PIFR have been identified (52). Moreover, HGS in male COPD patients exhibited a positive correlation with the EQ-5D utility score index, an indicator of quality of life (53).

Handgrip strength is closely associated with lower limb muscle function and serves as a meaningful indicator of overall limb strength across different age groups (54). Evidence suggests that grip strength may reflect nutritional status, with findings from the Hertfordshire Cohort Study indicating that healthier dietary patterns—characterized by prudent food choices, adequate dietary protein, antioxidants, vitamin D, and fatty fish consumption—are positively correlated with better grip strength (55). Furthermore, higher levels of physical activity and reduced sedentary behavior have been associated with greater grip strength (56, 57), underscoring the importance of lifestyle factors in maintaining muscular function. Additional determinants include ethnicity, age, sex, height, and socioeconomic status, with studies suggesting a heritability of approximately 52% for grip strength (58, 59).

Inspiratory muscle training may offer benefits for COPD patients with inspiratory muscle weakness (60). Although maximal inspiratory pressure is frequently reduced in COPD, only a minority of patients meet the criteria for profound weakness. While inspiratory muscle strength correlates with leg muscle strength, it does not directly associate with walking distance or symptom burden (61). Pulmonary obstruction in COPD significantly contributes to losses in muscle strength and mobility, and age-related declines in physical function may be influenced not only by reduced muscle strength and power but also by deteriorating pulmonary function (62). Quadriceps weakness is a recognized consequence of physical inactivity, particularly following hospitalizations for acute exacerbation of COPD (AECOPD). In such patients, progressive resistance training using ankle weights has been shown to be a feasible rehabilitation strategy even in severe cases (63). Impaired voluntary activation of the quadriceps is also common in advanced COPD and can be ameliorated with targeted exercise interventions (64).

Currently, it is estimated that 30–60% of COPD patients are affected by malnutrition, which can accelerate pulmonary cachexia and sarcopenia through progressive weight loss (65). The prevalence of cachexia increases with disease severity (66). Muscle atrophy and diaphragmatic dysfunction may further reduce exercise tolerance, creating a vicious cycle that worsens disease outcomes. In addition to the body mass index (BMI), more nuanced nutritional assessments are necessary, as BMI alone may underestimate malnutrition. Bioelectrical impedance analysis (BIA) is a non-invasive and practical method for body composition evaluation. It operates on the principle of electrical resistance through body tissues and provides estimates based on a three-compartment model (7, 67). The BIA can be applied across COPD disease stages and often reveals deficits in fat-free mass (FFM) and the PhA, which correlate with FEV₁, FFM, and prediction of mortality. Exacerbation frequency has also been linked to lower BMI and FFM (68), highlighting the importance of incorporating body composition and PhA assessment in COPD management.

Several studies report positive correlations between PhA and markers such as hemoglobin and albumin in hospitalized patients with cardiovascular disease, supporting the utility of PhA in identifying sarcopenia, malnutrition, and cachexia (69). Patients with lower PhA are typically older, at higher nutritional risk, and exhibit more pronounced malnutrition. They also exhibit unfavorable changes in anthropometric measures (e.g., weight, BMI, and arm and waist circumference), body composition parameters [e.g., skeletal muscle mass and appendicular skeletal mass index (ASMI)], and biochemical parameters (e.g., hemoglobin, albumin, and lipids) compared to those with normal PhA. Significant positive correlations have been observed between PhA and these nutritional metrics, indicating that lower PhA reflects poorer nutritional status (44, 45, 70).

An important consideration is that PhA is influenced by factors such as hydration, inflammation, and disease severity (67). While our models adjusted for key demographics and comorbidities, we could not include all potential confounders (71). However, this sensitivity is precisely what makes PhA a valuable composite biomarker (72). Evidence suggests that PhA integratively reflects the overall health of cell membranes and body cell mass, serving as a summary measure of the cumulative impact of various pathological processes (73). This includes the recent 2025 study (74) linking low PhA to computed tomography (CT)-based functional and pathological changes in smokers, the 2024 systematic review consolidating evidence on PhA’s association with disease severity, function, and prognosis in COPD, as well as prognostic studies (8, 70, 75–77). All statements regarding mortality and prognosis, especially in the Discussion, have been carefully reviewed and are currently specifically grounded in the findings from the COPD patient cohorts (43, 45, 78). Therefore, its persistent association with physical function, despite the potential influence of other variables, underscores its role as a robust, pragmatic indicator of global cellular health and functional status in COPD (79).

### Limitations

This study has several limitations. First, as a small, single-center investigation, the generalizability of the findings is limited, and future prospective randomized controlled trials are needed to confirm these results. Second, certain measures, such as walking speed and grip strength, are subject to human performance variability; and future studies would benefit from incorporating more objective biomechanical or device-based assessments. Finally, the relatively short observation period limits the evaluation of long-term relationships, highlighting the need for extended follow-up in subsequent research.

## Conclusion

PhA was identified as an independent predictor of both handgrip strength and knee extension strength in patients with COPD. These findings underscore the clinical relevance of PhA as a practical biomarker for identifying impaired muscle function and nutritional status in COPD, supporting its potential incorporation into routine assessment and management of individuals with COPD.

## Data Availability

The original contributions presented in the study are included in the article/supplementary material, further inquiries can be directed to the corresponding author.

## References

[1] Christenson SA Smith BM Bafadhel M Putcha N. Chronic obstructive pulmonary disease. Lancet. (2022) 399:2227–42. doi: 10.1016/S0140-6736(22)00470-6, 35533707

[2] Duffy SP Criner GJ. Chronic obstructive pulmonary disease: evaluation and management. Med Clin North Am. (2019) 103:453–61. doi: 10.1016/j.mcna.2018.12.005, 30955513

[3] Incalzi RA Scarlata S Pennazza G Santonico M Pedone C. Chronic obstructive pulmonary disease in the elderly. Eur J Intern Med. (2014) 25:320–8. doi: 10.1016/j.ejim.2013.10.001, 24183233

[4] Labaki WW Rosenberg SR. Chronic obstructive pulmonary disease. Ann Intern Med. (2020) 173:ITC17. doi: 10.7326/AITC202008040, 32745458

[5] Ritchie AI Wedzicha JA. Definition, causes, pathogenesis, and consequences of chronic obstructive pulmonary disease exacerbations. Clin Chest Med. (2020) 41:421–38. doi: 10.1016/j.ccm.2020.06.007, 32800196 PMC7423341

[6] Segal LN Martinez FJ. Chronic obstructive pulmonary disease subpopulations and phenotyping. J Allergy Clin Immunol. (2018) 141:1961–71. doi: 10.1016/j.jaci.2018.02.035, 29884286 PMC5996762

[7] Ward LC. Bioelectrical impedance analysis for body composition assessment: reflections on accuracy, clinical utility, and standardisation. Eur J Clin Nutr. (2019) 73:194–9. doi: 10.1038/s41430-018-0335-3, 30297760

[8] Murakami T Kobayashi T Ono H Shibuma H Tsuji K Nikkuni E . Phase angle as an indicator of sarcopenia and malnutrition in patients with chronic obstructive pulmonary disease. Respir Investig. (2024) 62:651–6. doi: 10.1016/j.resinv.2024.05.012, 38761479

[9] Lareau SC Fahy B Meek P Wang A. Chronic obstructive pulmonary disease (COPD). Am J Respir Crit Care Med. (2019) 199:P1–2. doi: 10.1164/rccm.1991P1, 30592446

[10] Rabe KF Watz H. Chronic obstructive pulmonary disease. Lancet. (2017) 389:1931–40. doi: 10.1016/S0140-6736(17)31222-9, 28513453

[11] Thibodeau JT Drazner MH. Bioelectrical impedance analysis: can it help in the assessment of congestion? J Card Fail. (2020) 26:24–5. doi: 10.1016/j.cardfail.2019.12.002, 31843568

[12] Popiolek-Kalisz J Szczygiel K. Bioelectrical impedance analysis and body composition in cardiovascular diseases. Curr Probl Cardiol. (2023) 48:101911. doi: 10.1016/j.cpcardiol.2023.101911, 37399855

[13] Ferguson CE Lambell KJ. Clinimetrics: bioelectrical impedance analysis in clinical practice. J Physiother. (2022) 68:280. doi: 10.1016/j.jphys.2022.05.007, 35715376

[14] Di Vincenzo O Marra M Di Gregorio A Pasanisi F Scalfi L. Bioelectrical impedance analysis (BIA)-derived phase angle in sarcopenia: a systematic review. Clin Nutr. (2021) 40:3052–61. doi: 10.1016/j.clnu.2020.10.048, 33183880

[15] Hamada R Tanabe N Oshima Y Yoshioka Y Maetani T Shiraishi Y . Phase angle measured by bioelectrical impedance analysis in patients with chronic obstructive pulmonary disease: associations with physical inactivity and frailty. Respir Med. (2024) 233:107778. doi: 10.1016/j.rmed.2024.107778, 39179050

[16] Catikkas NM Safer U. Bioelectrical impedance analysis: caution should be needed to avoid misinterpretation. Clin Nutr. (2023) 42:2289–90. doi: 10.1016/j.clnu.2023.09.018, 37775415

[17] Catikkas NM Safer U. The bioelectrical impedance analysis and sarcopenia: devices & standardization protocols. Clin Nutr. (2023) 42:2484–5. doi: 10.1016/j.clnu.2023.10.021, 38411020

[18] Gonzalez MC Heymsfield SB. Bioelectrical impedance analysis for diagnosing sarcopenia and cachexia: what are we really estimating? J Cachexia Sarcopenia Muscle. (2017) 8:187–9. doi: 10.1002/jcsm.12159, 28145079 PMC5377383

[19] Martins PC Gobbo LA Silva DAS. Bioelectrical impedance vector analysis (BIVA) in university athletes. J Int Soc Sports Nutr. (2021) 18:7. doi: 10.1186/s12970-020-00403-3, 33422070 PMC7796392

[20] Yang K Pan S Yang N Wu J Liu Y He Q. Effect of bioelectrical impedance technology on the prognosis of dialysis patients: a meta-analysis of randomized controlled trials. Ren Fail. (2023) 45:2203247. doi: 10.1080/0886022X.2023.2203247, 37133857 PMC10158555

[21] Foster KR Lukaski HC. Whole-body impedance—what does it measure? Am J Clin Nutr. (1996) 64:388s–96s. doi: 10.1093/ajcn/64.3.388S, 8780354

[22] Kyle UG Bosaeus I De Lorenzo AD Deurenberg P Elia M Gómez JM . Bioelectrical impedance analysis—part I: review of principles and methods. Clin Nutr. (2004) 23:1226–43. doi: 10.1016/j.clnu.2004.06.004, 15380917

[23] Barbosa-Silva MC Barros AJ Wang J Heymsfield SB Pierson RN Jr. Bioelectrical impedance analysis: population reference values for phase angle by age and sex. Am J Clin Nutr. (2005) 82:49–52. doi: 10.1093/ajcn/82.1.49 16002799

[24] Steiner MC Barton RL Singh SJ Morgan MDL. Bedside methods versus dual energy X-ray absorptiometry for body composition measurement in COPD. Eur Respir J. (2002) 19:626–31. doi: 10.1183/09031936.02.00279602, 11998990

[25] Schols AM Wouters EF Soeters PB Westerterp KR. Body composition by bioelectrical-impedance analysis compared with deuterium dilution and skinfold anthropometry in patients with chronic obstructive pulmonary disease. Am J Clin Nutr. (1991) 53:421–4. doi: 10.1093/ajcn/53.2.421, 1989407

[26] Agustí A Celli BR Criner GJ Halpin D Anzueto A Barnes P . Global initiative for chronic obstructive lung disease 2023 report: GOLD executive summary. Eur Respir J. (2023) 61:2300239. doi: 10.1183/13993003.00239-2023, 36858443 PMC10066569

[27] Chinese Experts Cystic Fibrosis Consensus Committee; Chinese Alliance for Rare Lung Diseases; Chinese Alliance for Rare Diseases, Bronchiectasis-China. Chinese experts consensus statement: diagnosis and treatment of cystic fibrosis (2023). Zhonghua Jie He He Hu Xi Za Zhi. (2023) 46:352–72. doi: 10.3760/cma.j.cn112147-20221214-00971 36990700

[28] Liu QX Zheng JP Xie YQ Guan WJ Jiang CY An JY . Single-breath and rebreathing methods for measurement of pulmonary diffusing function: a comparative study. Zhonghua Jie He He Hu Xi Za Zhi. (2013) 36:510–5. 24262087

[29] Cotes JE Dabbs JM Elwood PC Hall AM McDonald A Saunders MJ. Iron-deficiency anaemia: its effect on transfer factor for the lung (diffusiong capacity) and ventilation and cardiac frequency during sub-maximal exercise. Clin Sci. (1972) 42:325–35. doi: 10.1042/cs0420325 5013875

[30] Pulmonary Infection Assembly of Chinese Thoracic Society. Chinese expert consensus on the management of lower respiratory tract infections of *Pseudomonas aeruginosa* in adults (2022). Zhonghua Jie He He Hu Xi Za Zhi. (2022) 45:739–52. doi: 10.3760/cma.j.cn112147-20220407-00290 35927044

[31] Pulmonary Function and Clinical Respiratory Physiology Committee of Chinese Association of Chest Physicians; Chinese Thoracic Society; Pulmonary Function Group of Respiratory Branch of Chinese Geriatric Society. Standard technical specifications for methacholine chloride (methacholine) bronchial challenge test. Zhonghua Jie He He Hu Xi Za Zhi. (2024) 47:101–19. doi: 10.3760/cma.j.cn112147-20231019-00247 38309959

[32] Jiao P Wu F Wu J Sun Y Tian W Yu H . Surgical safety analysis and clinical experience sharing of myasthenia gravis patients aged 65 and over. Thorac Cancer. (2023) 14:717–23. doi: 10.1111/1759-7714.14799, 36691325 PMC10008675

[33] Wang H Tian Z Liu Y Li M Wang Q Zeng X . The clinical characteristics of systemic sclerosis-related pulmonary arterial hypertension. Zhonghua Nei Ke Za Zhi. (2014) 53:390–3. 25146407

[34] Wang JY Wang XY Sun HY. Study on the correlation between the pulmonary injury and the ET-1 serum level in ulcerative colitis patients. Zhongguo Zhong Xi Yi Jie He Za Zhi. (2012) 32:455–9. 22803421

[35] Brooks D Solway S Gibbons WJ. ATS statement on six-minute walk test. Am J Respir Crit Care Med. (2003) 167:1287. doi: 10.1164/ajrccm.167.9.950, 12714344

[36] Limpawattana P Putraveephong S Inthasuwan P Boonsawat W Theerakulpisut D Chindaprasirt J. Frailty syndrome in ambulatory patients with COPD. Int J Chron Obstruct Pulmon Dis. (2017) 12:1193–8. doi: 10.2147/COPD.S134233, 28458530 PMC5402886

[37] Balachandran VP Gonen M Smith JJ DeMatteo RP. Nomograms in oncology: more than meets the eye. Lancet Oncol. (2015) 16:e173–80. doi: 10.1016/S1470-2045(14)71116-7, 25846097 PMC4465353

[38] Huang YQ Liang CH He L Tian J Liang CS Chen X . Development and validation of a radiomics nomogram for preoperative prediction of lymph node metastasis in colorectal cancer. J Clin Oncol. (2016) 34:2157–64. doi: 10.1200/JCO.2015.65.9128, 27138577

[39] Guan Z Jin X Guan Z Liu S Tao K Luo L. The gut microbiota metabolite capsiate regulate SLC2A1 expression by targeting HIF-1α to inhibit knee osteoarthritis-induced ferroptosis. Aging Cell. (2023) 22:e13807. doi: 10.1111/acel.13807, 36890785 PMC10265160

[40] López-Campos JL Tan W Soriano JB. Global burden of COPD. Respirology. (2016) 21:14–23. doi: 10.1111/resp.12660, 26494423

[41] Orea-Tejeda A Gómez-Martínez M González-Islas D Flores-Cisneros L Keirns-Davis C Sánchez-Santillán R . The impact of hydration status and fluid distribution on pulmonary function in COPD patients. Sci Rep. (2022) 12:1216. doi: 10.1038/s41598-022-05192-0, 35075255 PMC8786821

[42] Zanella PB Àvila CC Chaves FC Gazzana MB Berton DC Knorst MM . Phase angle evaluation of lung disease patients and its relationship with nutritional and functional parameters. J Am Coll Nutr. (2021) 40:529–34. doi: 10.1080/07315724.2020.1801535, 32780649

[43] Martínez-Luna N Orea-Tejeda A González-Islas D Flores-Cisneros L Keirns-Davis C Sánchez-Santillán R . Association between body composition, sarcopenia and pulmonary function in chronic obstructive pulmonary disease. BMC Pulm Med. (2022) 22:106. doi: 10.1186/s12890-022-01907-1, 35346135 PMC8962175

[44] Custódio Martins P de Lima TR Silva AM Santos Silva DA. Association of phase angle with muscle strength and aerobic fitness in different populations: a systematic review. Nutrition. (2022) 93:111489. doi: 10.1016/j.nut.2021.111489, 34688022

[45] De Benedetto F Marinari S De Blasio F. Phase angle in assessment and monitoring treatment of individuals with respiratory disease. Rev Endocr Metab Disord. (2023) 24:491–502. doi: 10.1007/s11154-023-09786-5, 36694055

[46] Bernardi E Pomidori L Bassal F Contoli M Cogo A. Respiratory muscle training with normocapnic hyperpnea improves ventilatory pattern and thoracoabdominal coordination, and reduces oxygen desaturation during endurance exercise testing in COPD patients. Int J Chron Obstruct Pulmon Dis. (2015) 10:1899–906. doi: 10.2147/COPD.S88609, 26392764 PMC4573075

[47] Celis-Morales CA Welsh P Lyall DM Steell L Petermann F Anderson J . Associations of grip strength with cardiovascular, respiratory, and cancer outcomes and all cause mortality: prospective cohort study of half a million UK biobank participants. BMJ. (2018) 361:k1651. doi: 10.1136/bmj.k1651, 29739772 PMC5939721

[48] Strand BH Cooper R Bergland A Jørgensen L Schirmer H Skirbekk V . The association of grip strength from midlife onwards with all-cause and cause-specific mortality over 17 years of follow-up in the Tromsø study. J Epidemiol Community Health. (2016) 70:1214–21. doi: 10.1136/jech-2015-206776, 27229009 PMC5136688

[49] Ortega FB Silventoinen K Tynelius P Rasmussen F. Muscular strength in male adolescents and premature death: cohort study of one million participants. BMJ. (2012) 345:e7279. doi: 10.1136/bmj.e7279 23169869 PMC3502746

[50] Leong DP Teo KK Rangarajan S Lopez-Jaramillo P Avezum A Jr Orlandini A . Prognostic value of grip strength: findings from the Prospective Urban Rural Epidemiology (PURE) study. Lancet. (2015) 386:266–73. doi: 10.1016/S0140-6736(14)62000-6, 25982160

[51] Qiu P Chen M Lv S Xie J Wu J. The association between walking pace and hand grip strength with the risk of chronic obstructive pulmonary disease: a bidirectional mendelian randomization study. BMC Pulm Med. (2023) 23:450. doi: 10.1186/s12890-023-02759-z, 37986176 PMC10658936

[52] Suriyakul A Saiphoklang N Barjaktarevic I Cooper CB. Correlation between hand grip strength and peak inspiratory flow rate in patients with stable chronic obstructive pulmonary disease. Diagnostics. (2022) 12:3050. doi: 10.3390/diagnostics12123050, 36553057 PMC9777131

[53] Lee SH Kim SJ Han Y Ryu YJ Lee JH Chang JH. Hand grip strength and chronic obstructive pulmonary disease in Korea: an analysis in KNHANES VI. Int J Chron Obstruct Pulmon Dis. (2017) 12:2313–21. doi: 10.2147/COPD.S142621, 28831248 PMC5552152

[54] Bohannon RW Magasi SR Bubela DJ Wang YC Gershon RC. Grip and knee extension muscle strength reflect a common construct among adults. Muscle Nerve. (2012) 46:555–8. doi: 10.1002/mus.23350, 22987697 PMC3448119

[55] Norman K Stobäus N Gonzalez MC Schulzke JD Pirlich M. Hand grip strength: outcome predictor and marker of nutritional status. Clin Nutr. (2011) 30:135–42. doi: 10.1016/j.clnu.2010.09.010, 21035927

[56] Hamer M Stamatakis E. Screen-based sedentary behavior, physical activity, and muscle strength in the English longitudinal study of ageing. PLoS One. (2013) 8:e66222. doi: 10.1371/journal.pone.0066222, 23755302 PMC3670922

[57] Gianoudis J Bailey CA Daly RM. Associations between sedentary behaviour and body composition, muscle function and sarcopenia in community-dwelling older adults. Osteoporos Int. (2015) 26:571–9. doi: 10.1007/s00198-014-2895-y, 25245026

[58] Newman AB Haggerty CL Goodpaster B Harris T Kritchevsky S Nevitt M . Strength and muscle quality in a well-functioning cohort of older adults: the health, aging and body composition study. J Am Geriatr Soc. (2003) 51:323–30. doi: 10.1046/j.1532-5415.2003.51105.x 12588575

[59] Ramlagan S Peltzer K Phaswana-Mafuya N. Hand grip strength and associated factors in non-institutionalised men and women 50 years and older in South Africa. BMC Res Notes. (2014) 7:8. doi: 10.1186/1756-0500-7-8, 24393403 PMC3892054

[60] Janaudis-Ferreira T Wadell K Sundelin G Lindström B. Thigh muscle strength and endurance in patients with COPD compared with healthy controls. Respir Med. (2006) 100:1451–7. doi: 10.1016/j.rmed.2005.11.001, 16337114

[61] Kofod LM Hage T Christiansen LH Skalkam K Martinez G Godtfredsen NS . Inspiratory muscle strength and walking capacity in patients with COPD. Eur Clin Respir J. (2020) 7:1700086. doi: 10.1080/20018525.2019.1700086, 31853341 PMC6913623

[62] Sillanpää E Stenroth L Bijlsma AY Rantanen T McPhee JS Maden-Wilkinson TM . Associations between muscle strength, spirometric pulmonary function and mobility in healthy older adults. Age. (2014) 36:9667. doi: 10.1007/s11357-014-9667-7, 25073451 PMC4150884

[63] Kofod LM Døssing M Steentoft J Kristensen MT. Resistance training with ankle weight cuffs is feasible in patients with acute exacerbation of COPD. J Cardiopulm Rehabil Prev. (2017) 37:49–56. doi: 10.1097/HCR.0000000000000230, 28005680

[64] Vivodtzev I Flore P Lévy P Wuyam B. Voluntary activation during knee extensions in severely deconditioned patients with chronic obstructive pulmonary disease: benefit of endurance training. Muscle Nerve. (2008) 37:27–35. doi: 10.1002/mus.20867, 17912747

[65] Kaluźniak-Szymanowska A Krzymińska-Siemaszko R Deskur-Śmielecka E Lewandowicz M Kaczmarek B Wieczorowska-Tobis K. Malnutrition, sarcopenia, and malnutrition-sarcopenia syndrome in older adults with COPD. Nutrients. (2021) 14:44. doi: 10.3390/nu14010044, 35010919 PMC8746722

[66] Sepúlveda-Loyola W Osadnik C Phu S Morita AA Duque G Probst VS. Diagnosis, prevalence, and clinical impact of sarcopenia in COPD: a systematic review and meta-analysis. J Cachexia Sarcopenia Muscle. (2020) 11:1164–76. doi: 10.1002/jcsm.12600, 32862514 PMC7567149

[67] Norman K Stobäus N Pirlich M Bosy-Westphal A. Bioelectrical phase angle and impedance vector analysis—clinical relevance and applicability of impedance parameters. Clin Nutr. (2012) 31:854–61. doi: 10.1016/j.clnu.2012.05.008, 22698802

[68] de Blasio F de Blasio F Miracco Berlingieri G Bianco A la Greca M Franssen F . Evaluation of body composition in COPD patients using multifrequency bioelectrical impedance analysis. Int J Chron Obstruct Pulmon Dis. (2016) 11:2419–26. doi: 10.2147/COPD.S110364, 27757027 PMC5053371

[69] Hirose S Nakajima T Nozawa N Katayanagi S Ishizaka H Mizushima Y . Phase angle as an Indicator of sarcopenia, malnutrition, and Cachexia in inpatients with cardiovascular diseases. J Clin Med. (2020) 9:2554. doi: 10.3390/jcm9082554, 32781732 PMC7463846

[70] Jin X Yang Y Chen G Shao Y Liu C Li R . Correlation between body composition and disease severity in patients with chronic obstructive pulmonary disease. Front Med. (2024) 11:1304384. doi: 10.3389/fmed.2024.1304384, 38549868 PMC10972851

[71] Valisoltani N Mohammadi H Aliannejad R Naeini F Harsini AR Sadeghi E . Association of phase angle with sarcopenia and muscle function in patients with COPD: a case-control study. BMC Pulm Med. (2024) 24:18. doi: 10.1186/s12890-023-02814-9, 38184558 PMC10771663

[72] Lukaski H Raymond-Pope CJ. New Frontiers of body composition in sport. Int J Sports Med. (2021) 42:588–601. doi: 10.1055/a-1373-5881, 33621995 PMC8421000

[73] Stobäus N Pirlich M Valentini L Schulzke JD Norman K. Determinants of bioelectrical phase angle in disease. Br J Nutr. (2012) 107:1217–20. doi: 10.1017/S0007114511004028, 22309898

[74] Xia XX Li CX Xue XX Chen YJ He F Guo HR. Association between phase angle and all-cause mortality in adults aged 18–49 years: NHANES 1999-2004. Sci Rep. (2025) 15:2785. doi: 10.1038/s41598-025-86825-y, 39843978 PMC11754445

[75] Kobayashi T Murakami T Ono H Takahashi T. Phase angle as an indicator of physical activity in patients with stable chronic obstructive pulmonary disease. Nutrition. (2024) 120:112330. doi: 10.1016/j.nut.2023.112330, 38262195

[76] Kobayashi T Murakami T Ono H Togashi S Takahashi T. Segmental phase angle can predict incidence of severe exacerbation in male patients with COPD. Nutrition. (2025) 132:112681. doi: 10.1016/j.nut.2024.112681, 39826429

[77] Phantayuth D Chuaychoo B Supaporn S Nana A Ramyarangsi P Ajjimaporn A. Effectiveness of a 12-week combining tai chi and yoga program on pulmonary function and functional fitness in COPD patients. Respir Med. (2024) 234:107842. doi: 10.1016/j.rmed.2024.107842, 39433109

[78] Gómez-Martínez M Rodríguez-García W González-Islas D Orea-Tejeda A Keirns-Davis C Salgado-Fernández F . Impact of body composition and sarcopenia on mortality in chronic obstructive pulmonary disease patients. J Clin Med. (2023) 12:1321. doi: 10.3390/jcm12041321, 36835862 PMC9967244

[79] Xie AN Huang WJ Ko CY. Extracellular water ratio and phase angle as predictors of exacerbation in chronic obstructive pulmonary disease. Adv Respir Med. (2024) 92:230–40. doi: 10.3390/arm92030023, 38921062 PMC11200775

